# Key considerations to promote digital inclusivity within healthcare: stakeholders’ and under‐served groups perspectives

**DOI:** 10.1002/alz70860_100787

**Published:** 2025-12-23

**Authors:** Sarah Wilson, Clare Tolley, Ríona Mc Ardle, Robert Slight, Sarah Slight

**Affiliations:** ^1^ Newcastle University, Newcastle Upon Tyne, United Kingdom; ^2^ Newcastle University, Newcastle Upon Tyne, Tyne and Wear, United Kingdom; ^3^ Newcastle University, Newcastle upon Tyne, Tyne and Wear, United Kingdom

## Abstract

**Background:**

Digital Health Technologies (DHTs) have the potential to assist with the early detection of dementia‐causing diseases by identifying and monitoring subtle changes in signs and symptoms prior to cognitive decline. However, under‐served groups often face digital exclusion, as they are unable to access, use or have the motivation to use DHTs. To advance digital health equity, this study explored the specific challenges facing underserved groups and how they may be overcome, and stakeholders’ perspectives on the key considerations to promoting digital inclusivity within healthcare.

**Method:**

Two qualitative studies were conducted. One with under‐served individuals who represented at least two CLEARS (Culture, Limiting conditions, low Educational attainment, older Age, Residence, low Socioeconomic status) domains,^(1)^ and the second study with stakeholders, including health commissioners, who have a professional interest in supporting these groups. Under‐served groups were recruited through community organisations in North‐East England, and stakeholders were recruited via purposeful snowball sampling and social media. Using semi‐structured interviews and focus groups, we explored current strategies to support digital inclusion. A reflexive thematic analysis, assisted by Nvivo (QSR, v15), was conducted to identify key themes, which were further refined with the research team.

**Result:**

Twenty‐nine under‐served individuals and 17 stakeholders from various professional backgrounds participated. Four key considerations emerged and centred around supportive networks, co‐design approaches, digital skills education, and raising awareness. Participants perceived cross‐organizational collaboration as vital to providing free devices and connectivity due to the resources required. They also recommended co‐design to ensure DHTs are tailored to users' needs. Under‐served groups expressed concerns about relying on social networks for digital support, having experienced controlling behaviours. Both groups advocated for increased funding for educational services and staff training. Non‐digital, community‐based advertising could help raise awareness of the benefits of DHTs and available support.

**Conclusion:**

These findings can guide researchers, developers, and healthcare professionals in advancing digital health equity for under‐served groups. Further research should explore additional considerations for those with health conditions or cognitive decline.

1) Wilson, S., et al. (2024) Who is most at risk of digital exclusion within healthcare?, *International Journal of Pharmacy Practice*, 32, Supplement_1, i3–i4

